# Supplementary material for: Whole blood RNA-seq demonstrates an increased host immune response in individuals with cystic fibrosis who develop nontuberculous mycobacterial pulmonary disease
Source: PLoS One. 2022 Dec 8;17(12):e0278296. doi: 10.1371/journal.pone.0278296 (PMC9731410; doi:10.1371/journal.pone.0278296)
Supplement: S1 Table — (DOCX) [file pone.0278296.s001.docx]

**S1 Table. Demographic and clinical characteristics of patients infected with MAC or MABs at time of first positive NTM culture**

|  | MAC  (n = 21) | MABs  (n = 12) |
| --- | --- | --- |
| Progression to NTM-PD – n (%) | 6 (28.6%) | 5 (41.7%) |
| Age - median (IQR) | 27.0 (22 - 39) | 31.5 (22 - 38) |
| Females - n (%) | 6 (28.6%) | 3 (25%) |
| Genotype - n (%) |  |  |
| F508del/F508del  F508del/other  Others | 8 (38.1%)  9 (42.9%)  4 (19.0%) | 4 (33.0%)  6 (50.0%)  2 (16.7%) |
| Pancreatic insufficiency - n (%) | 15 (71.4%) | 10 (83.3%) |
| CF diabetes - n (%) ^a^ | 5 (23.8%) | 2 (16.7%) |
| Body mass index - median (IQR) | 22.0 (21 - 23) | 21.3 (20 - 23) |
| FEV_1_ [% predicted] – median (IQR) | 80.6 (73 - 95) | 67.5 (54 - 84) |
| Oral steroids exposure - n (%) | 1 (4.7%) | 1 (8.3%) |
| Macrolide exposure - n (%) | 7 (33.3%) | 1 (8.33%) |

**MAC.** Mycobacterium avium complex. **MABs.** Mycobacterium abscessus complex. **NTM-PD.** Nontuberculous mycobacteria pulmonary disease. ^a^ missing data for 1 patient infected with MABs. No significant differences were found in any variable using univariate statistics, unpaired Wilcoxon rank-sum or Fisher test respectively.
