# Supplementary material for: Whole blood RNA-seq demonstrates an increased host immune response in individuals with cystic fibrosis who develop nontuberculous mycobacterial pulmonary disease
Source: PLoS One. 2022 Dec 8;17(12):e0278296. doi: 10.1371/journal.pone.0278296 (PMC9731410; doi:10.1371/journal.pone.0278296)
Supplement: S2 Table — (DOCX) [file pone.0278296.s002.docx]

**S2 Table. Gene set enrichment analysis of whole blood gene expression in those who did vs. did not develop NTM-PD, excluding 6 samples taken after diagnosis of NTM-PD.**

| **Pathway** | **p-value** | **FDR** | **NES** | **Size** |
| --- | --- | --- | --- | --- |
| Interferon α response | < 0.0001 | < 0.0001 | 2.9858 | 96 |
| Interferon γ response | < 0.0001 | < 0.0001 | 2.5549 | 199 |
| Heme metabolism | < 0.0001 | < 0.0001 | 1.8731 | 188 |
| G2M checkpoint | < 0.0001 | 0.0002 | 1.6814 | 192 |
| Mitotic spindle | < 0.0001 | 0.0002 | 1.6905 | 189 |
| IL-6-JAK-STAT3 signaling | 0.0002 | 0.0016 | 1.7723 | 81 |
| Protein secretion | 0.0005 | 0.0029 | 1.6951 | 86 |
| Tumor necrosis factor-α signaling via NFK-β | 0.0004 | 0.0029 | 1.4885 | 198 |
| Adipogenesis | 0.0006 | 0.0031 | 1.5041 | 192 |

Enrichment of molecular signatures database 3.0, accessed in May 2022. **NES:** normalized enrichment score, positive scores represent enrichment in patients that developed NTM-PD. **FDR:** false discovery rate. **Size** specifies the number of total features in the pathway
