# Supplementary figures and images for: Whole blood RNA-seq demonstrates an increased host immune response in individuals with cystic fibrosis who develop nontuberculous mycobacterial pulmonary disease
Source: PLoS One. 2022 Dec 8;17(12):e0278296. doi: 10.1371/journal.pone.0278296 (PMC9731410; doi:10.1371/journal.pone.0278296)

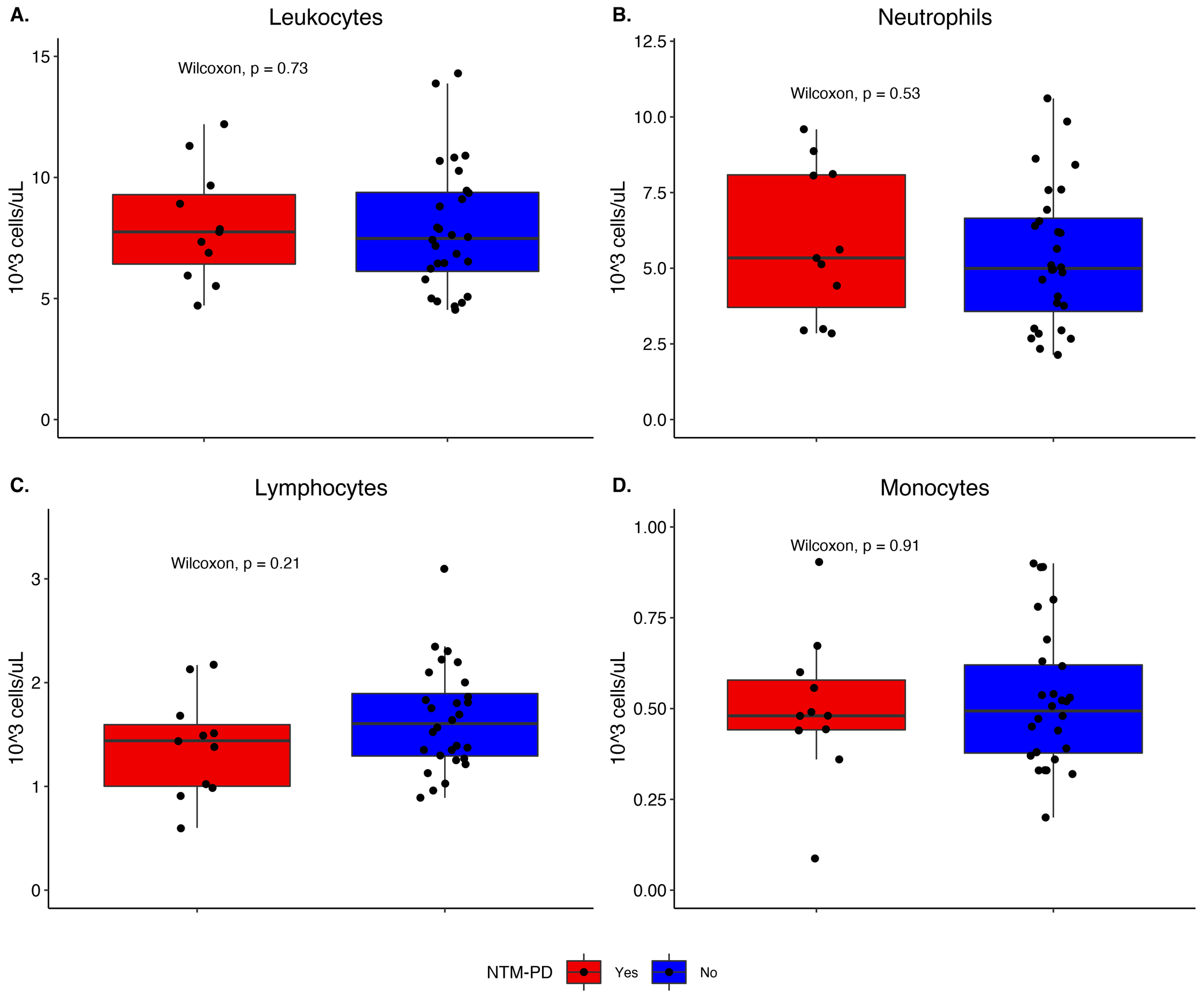

Supplement: S1 Fig — Boxplots with the distribution of absolute values for leukocytes, neutrophils, lymphocytes, and monocytes in complete blood counts at the time of blood sample procurement. No significant differences using the unpaired Wilcoxon rank-sum test. (TIF) [file pone.0278296.s003.tif]

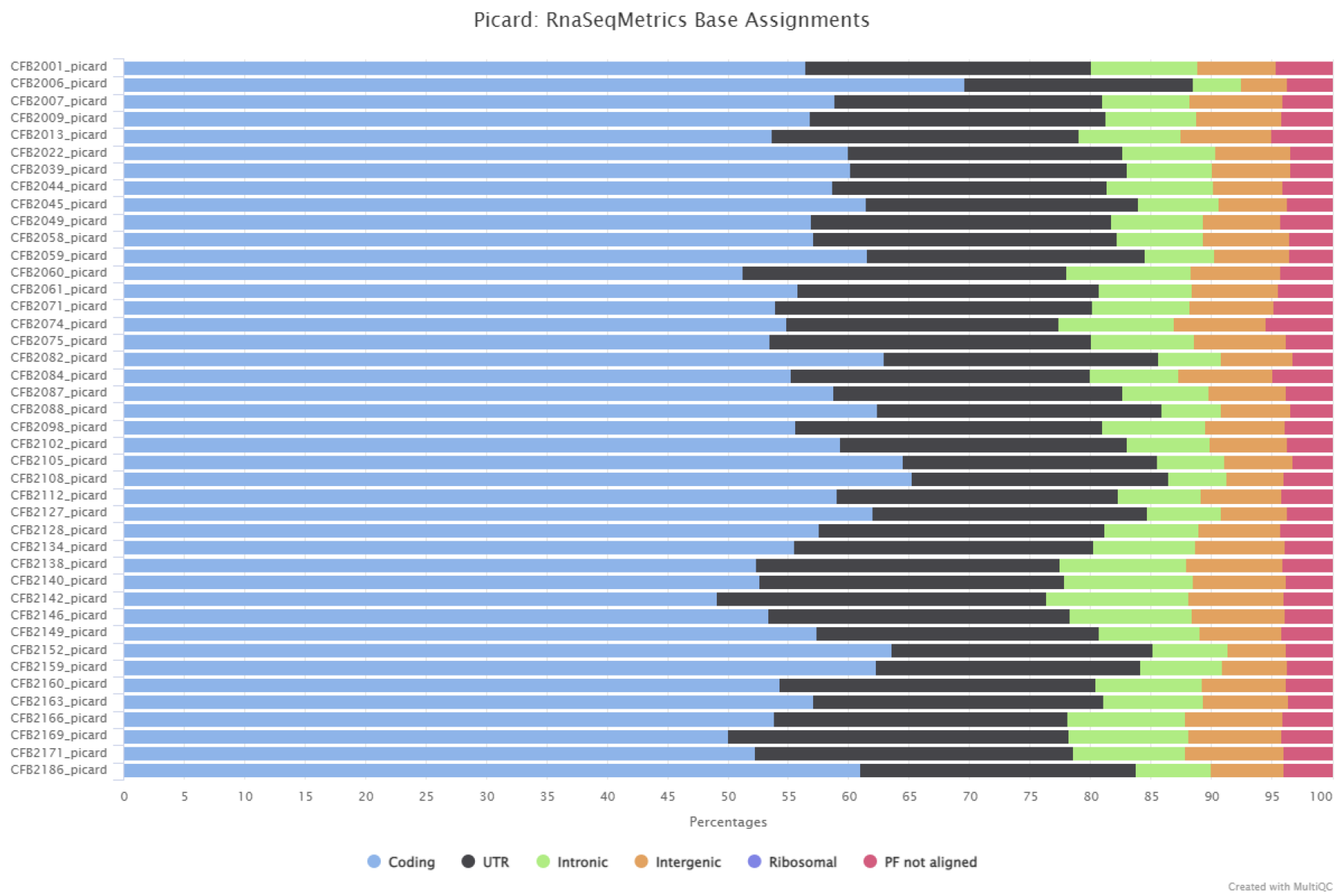

Supplement: S2 Fig — Coding: protein-coding region. UTR: untranscribed region. Intronic: intronic region. Intergenic: intergenic region. Ribosomal: mapping to ribosomal RNA or proteins. PF not aligned: bases that passed the quality filter and were not aligned. (TIF) [file pone.0278296.s004.tif]

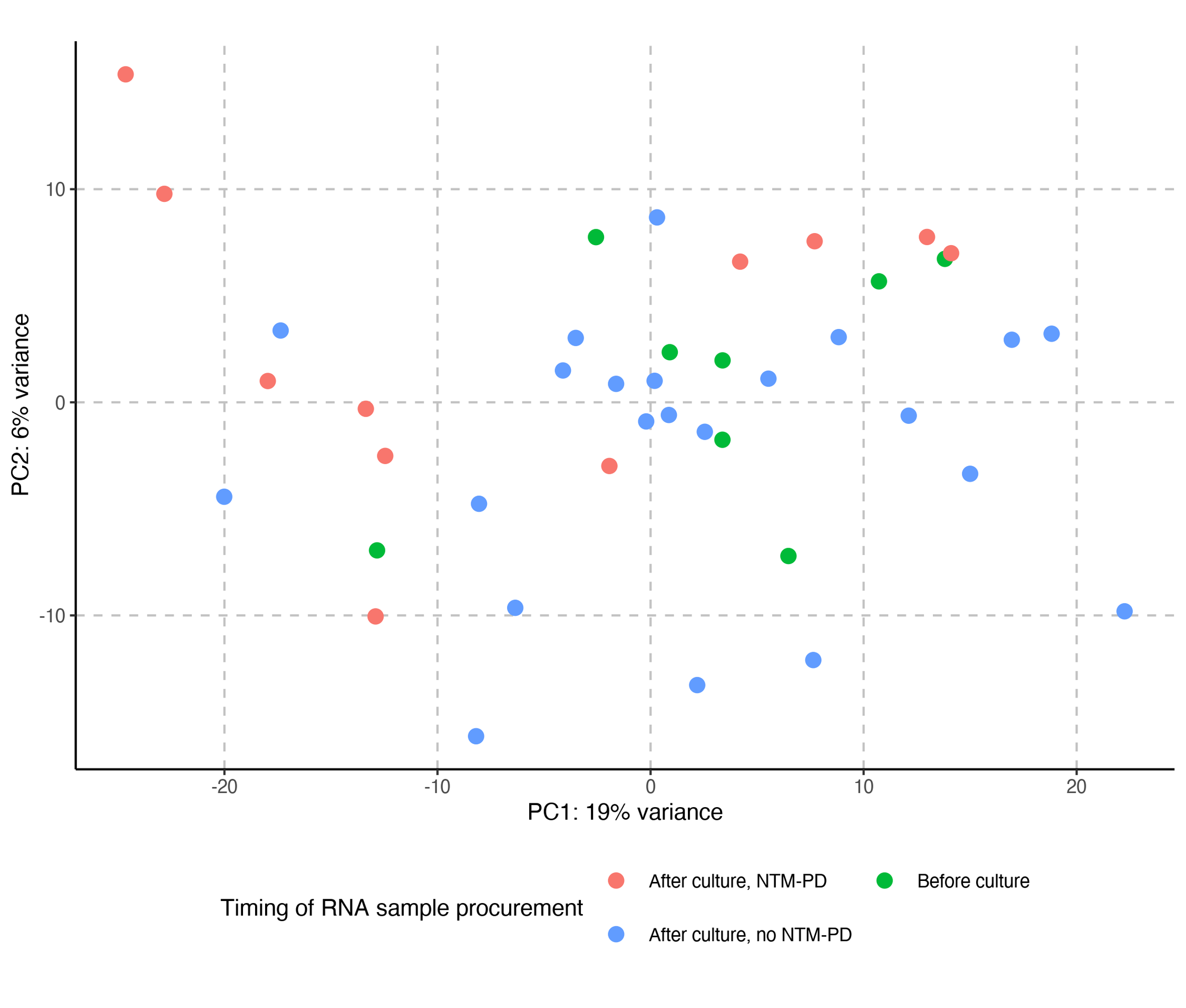

Supplement: S3 Fig — Separated by the timing of blood sample procurement for RNAseq into three categories: before first positive NTM culture, after first positive NTM culture without progression to NTM-PD, and after first positive NTM culture with progression to NTM-PD. No apparent separation is seen graphically. A PermANOVA test shows no significant differences in the distance matrices by group (p = 0.29). (TIFF) [file pone.0278296.s005.tiff]

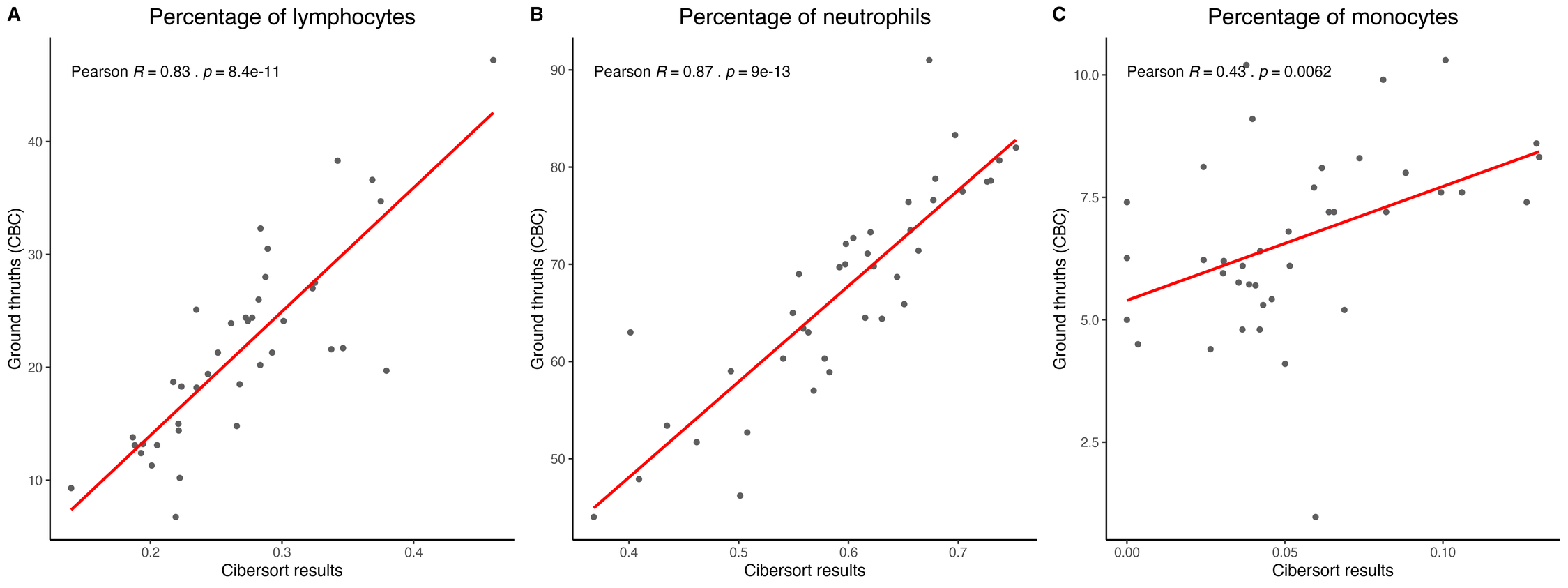

Supplement: S4 Fig — (TIF) [file pone.0278296.s006.tif]

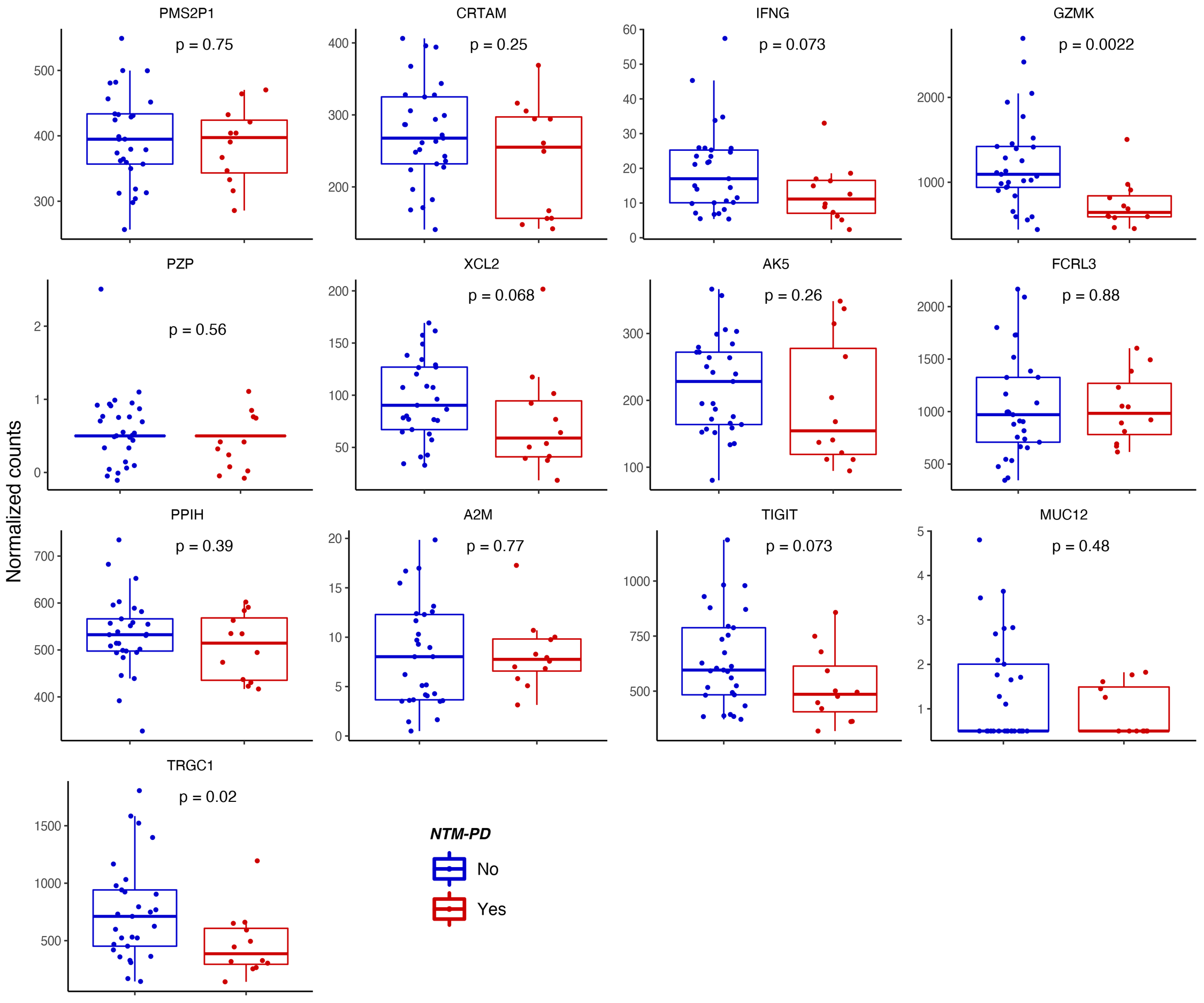

Supplement: S5 Fig — The p-values represent the results of independent Wilcoxon tests. The feature TRGC1 (T Cell Receptor Gamma Constant 1) was a duplicated probe in the initial analysis, while the uncharacterized locus FLJ45825 was not detected in our dataset. PMS2P1: PMS1 homolog 2, Mismatch Repair System Component Pseudogene 1. CRTAM: Cytotoxic and regulatory T cell Molecule. IFNG: Interferon Gamma. GZMK: Granzyme K. PZP: PZP alpha-2-macroglobulin like. XCL2: X-C motif chemokine ligand 2. AK5: Adenylate kinase 5. FCRL3: Fc receptor Like 3. PPIH: Peptidylprolyl isomerase H. A2M: Alpha-2-macroglobulin. TIGIT: T cell immunoreceptor with Ig and ITIM domains. MUC12: Mucin 12, cell surface associated. (TIF) [file pone.0278296.s007.tif]
